# Mechanistic Insights Expatiating the Redox-Active-Metal-Mediated Neuronal Degeneration in Parkinson’s Disease

**DOI:** 10.3390/ijms23020678

**Published:** 2022-01-08

**Authors:** Tapan Behl, Piyush Madaan, Aayush Sehgal, Sukhbir Singh, Md Khalid Anwer, Hafiz A. Makeen, Mohammed Albratty, Syam Mohan, Simona Bungau

**Affiliations:** 1 Chitkara College of Pharmacy, Chitkara University, Rajpura 140401, Punjab, India; piyushmadaan4811@gmail.com (P.M.); aayushsehgal00@gmail.com (A.S.); sukhbir.singh@chitkara.edu.in (S.S.); 2 Department of Pharmaceutics, College of Pharmacy, Prince Sattam Bin Abdulaziz University, Alkharj 16278, Saudi Arabia; m.anwer@psau.edu.sa; 3 Pharmacy Practice Research Unit, Clinical Pharmacy Department of College of Pharmacy, Jazan University, Jazan 45142, Saudi Arabia; hafiz@jazanu.edu.sa; 4 Department of Pharmaceutical Chemistry, College of Pharmacy, Jazan University, Jazan 45142, Saudi Arabia; malbratty@jazanu.edu.sa; 5 Substance Abuse and Toxicology Research Center, Jazan University, Jazan 45142, Saudi Arabia; syammohanm@yahoo.com; 6 School of Health Sciences, University of Petroleum and Energy Studies, Dehradun 248007, Uttarakhand, India; 7 Department of Pharmacy, Faculty of Medicine and Pharmacy, University of Oradea, 410028 Oradea, Romania

**Keywords:** neurodegenerative malady, iron, copper, oxidative stress, α-synuclein aggregation, Parkinson’s disease

## Abstract

Parkinson’s disease (PD) is a complicated and incapacitating neurodegenerative malady that emanates following the dopaminergic (DArgic) nerve cell deprivation in the substantia nigra pars compacta (SN-PC). The etiopathogenesis of PD is still abstruse. Howbeit, PD is hypothesized to be precipitated by an amalgamation of genetic mutations and exposure to environmental toxins. The aggregation of α-synucelin within the Lewy bodies (LBs), escalated oxidative stress (OS), autophagy-lysosome system impairment, ubiquitin-proteasome system (UPS) impairment, mitochondrial abnormality, programmed cell death, and neuroinflammation are regarded as imperative events that actively participate in PD pathogenesis. The central nervous system (CNS) relies heavily on redox-active metals, particularly iron (Fe) and copper (Cu), in order to modulate pivotal operations, for instance, myelin generation, synthesis of neurotransmitters, synaptic signaling, and conveyance of oxygen (O_2_). The duo, namely, Fe and Cu, following their inordinate exposure, are viable of permeating across the blood–brain barrier (BBB) and moving inside the brain, thereby culminating in the escalated OS (through a reactive oxygen species (ROS)-reliant pathway), α-synuclein aggregation within the LBs, and lipid peroxidation, which consequently results in the destruction of DArgic nerve cells and facilitates PD emanation. This review delineates the metabolism of Fe and Cu in the CNS, their role and disrupted balance in PD. An in-depth investigation was carried out by utilizing the existing publications obtained from prestigious medical databases employing particular keywords mentioned in the current paper. Moreover, we also focus on decoding the role of metal complexes and chelators in PD treatment. Conclusively, metal chelators hold the aptitude to elicit the scavenging of mobile/fluctuating metal ions, which in turn culminates in the suppression of ROS generation, and thereby prelude the evolution of PD.

## 1. Introduction

Parkinson’s disease (PD) is one of the most perpetual, elaborative, multifaceted, and disabling neurodegenerative maladies with an expeditiously escalating prevalence and economic encumbrance on mankind in the context of medical intervention [1,2,3]. Over the past 10 years, there has been prodigious corroboration indicating that PD is an intricate multifactorial malady that encompasses both motor and non-motor manifestations [4]. PD is delineated through four pivotal manifestations, namely, bradykinesia, rigor, tremor, and postural impairment, which in particular are precipitated through dopaminergic (DArgic) nerve cell deprivation in the substantia nigra pars compacta (SN-PC) [5,6]. PD is hugely infrequent in persons falling beneath the age range of 35–40 years, while it affects approximately 1–2% of persons falling beyond the age range of 60–65 years and is more likely to emerge in persons falling beyond the age range of 80–85 years across the nation [7]. According to recent studies, men exhibit nearly twofold greater susceptibility to developing PD in comparison to women. This could be due to the escalation in the neuronal protection provided by elevated levels of dopamine (DA) in the striatal region of women, presumably associated with estrogen activity [8,9,10,11,12,13]. Every individual experiencing PD possesses distinctive therapeutic outcomes, difficulties, and prognosis [4]. There has been a substantial breakthrough in comprehending the neuropathology of PD, its evolution across the entire central nervous system (CNS), pathways and alterations that underpin the disease and its manifestations [14]. Owing to the intricate and perplexing nature of the malady, the etiopathogenesis of PD is still inexplicit. However, genetic mutations and environmental toxin exposure in combination are considered to partake in the disease etiology [15]. An expanding body of pre-clinical corroboration demonstrates the intricate interrelationship between α-synuclein aggregation within the Lewy bodies (LBs), oxidative stress (OS), autophagy-lysosome system impairment, ubiquitin-proteasome system (UPS) impairment, mitochondrial abnormality, inflammation of nerve cells, and programmed cell death as imperative pathways that underlie the disease pathogenesis [15,16,17]. Present-day therapeutic approaches mainly focus on DA replacement therapy by employing a DA precursor, namely, Levodopa (L-dopa). Although L-dopa-assisted therapy can render symptomatic relief or upgrade the quality of well-being, no therapy has been depicted to terminate the evolution of the malady [18,19,20].

Metal ions have been reported to be actively engaged in several biological operations of both eukaryotic and prokaryotic organisms [21]. Although the alkali metals (group one metals), for instance, sodium (Na) and potassium (K), and alkaline-earth metals (group two metals), for instance, calcium (Ca) and magnesium (Mg), pertain to the group of highly profuse physiological metal ions, the transition metals, for instance, iron (Fe), copper (Cu), Zinc (Zn), cobalt (Co), and nickel (Ni), etc., are employed in numerous pivotal physiological operations, such as respiration, photosynthesis, and several other operations crucial to cellular biotransformation. The transition metals, namely, Fe and Cu, exist as the most plenteous redox-active metals. An individual’s body comprises nearly 5 g of Fe, which is present in physiological systems in three different forms, viz., ferrous, ferric, and ferryl, possessing +2 (Fe^+2^), +3 (Fe^+3^), and +4 (Fe^+4^) oxidation numbers, respectively, with the latter one being infrequent [21]. On the other hand, Cu is present in physiological systems in two fundamental forms, namely, cuprous and cupric, possessing oxidation states +1 (Cu^+1^) and +2 (Cu^+2^), respectively [21]. The two, namely, Fe and Cu, are capable of occupying many valence states in proteins, and further trigger the activation of oxygen (O_2_), which is employed by numerous biocatalysts implicated in cellular respiration. Contrarily, up-regulation of redox-active metals contributes to their escalated interaction with O_2_, which eventually gives rise to reactive oxygen species (ROS) generation, which might be deleterious to organisms under specific circumstances and partake in cellular deterioration at numerous stages, for instance, deoxyribonucleic acid (DNA), protein (α-synuclein), and membrane lipids (glycolipids, phospholipids, and cholesterol) [22]. The proportion of the CNS, namely, the brain, possesses exorbitant oxidative biotransformation operations and comparatively low antioxidant safeguarding, and as a result, it is peculiarly exposed to OS, which could be aggravated via metals of redox-active nature that are widely diffused within the brain, for instance, Fe and Cu [23,24,25].

**Figure 1:**
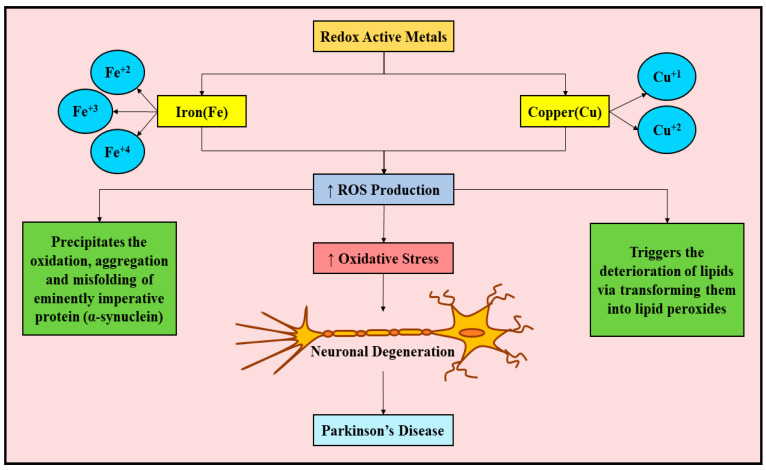
Redox-active metals (Fe, and Cu), their oxidation states, and their implications in Parkinson’s disease via ROS-mediated pathway. Fe exists in three oxidation states, namely, Fe^+2^, Fe^+3^, and Fe^+4^. On the other hand, Cu exists in two oxidation states, namely, Cu^+1^ and Cu^+2^. Escalation in the Fe and Cu levels can contribute to increased ROS production, which in turn culminates in increased oxidative stress, oxidation, aggregation and misfolding of critical protein (α-synuclein), and deterioration of lipids via transforming them into lipid peroxides. These processes in combination lead to nerve cell degeneration, and eventually culminate in Parkinson’s disease evolution. Fe, iron; Fe^+2^, ferrous Fe; Fe^+3^, ferric Fe; Fe^+4^, ferryl Fe; Cu, copper; Cu^+1^, cuprous form of Cu; Cu^+2^, cupric form of Cu; ROS, reactive oxygen species; ↑, increased/elevated.

Fe and Cu have been reported to be actively engaged in the regulation of cardinal operations of the CNS, namely, generation of myelin, synthesis of neurotransmitters, synaptic signaling, and conveyance of O_2_ [26,27]. The aforementioned operations are accomplished via the structural and catalytic modulation of protein channels, biocatalysts/enzymes, and receptors [26,27,28]. Cu and Fe’s redox characteristics are tremendously essential for the majority of the above-described biological operations; still, both serve as producers of ROS. ROS precipitates nerve cell degeneration via prompting the oxidation, accumulation, and misfolding of extremely important protein (α-synuclein). Additionally, ROS can also trigger the deterioration of lipids via transforming them into lipid peroxides, which constitute critical participants in the Fe-reliant type of cell death affecting nerve cells (ferroptosis) [29]. Figure 1 depicts the redox-active metals (Fe, and Cu), their oxidation states, and their implications in PD via the ROS-mediated pathway.

Cells exhibit multiple pathways to restore or regulate oxidative destruction and scavenge unpaired electrons containing molecules termed free radicals, so as to impede OS and successive cell destruction. The CNS possesses a number of tiny molecules and proteins that carry out the appropriate storage, conveyance, and delivery of Fe and Cu to the desired site, thereby precluding Fe and Cu-effectuated ROS generation. It has been reported that the brains of mice and human beings exhibit a buildup of both Fe and Cu as they experience ageing, implying that their modulation is immensely impacted by age-reliant processes [30]. This disruption in the equilibrium is enormously prominent in several incapacitating neurodegenerative maladies, for instance, PD, amyotrophic lateral sclerosis (ALS), and Alzheimer’s disease (AD). The current review focuses on delineating the metabolism of metals of redox-active nature (specifically Fe and Cu) within the CNS, their role, disrupted equilibrium in PD, and therapeutic opportunities intended to control these vital physiologically active metals in order to alleviate or impede nerve cell degeneration.

## 2. Parkinson’s Disease: Etiological Factors, Pathogenic Events, and Treatment

**Figure 2:**
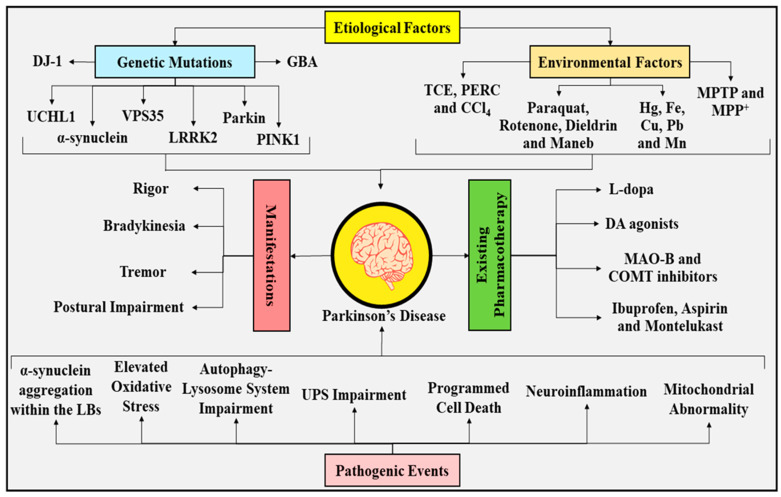
Current understanding of the manifestations, etiological factors, pathogenic events, and existing pharmacotherapy of Parkinson’s disease. *DJ-1*, *protein deglycase*; *UCHL1*, *ubiquitin carboxy* (*C*)*-terminal hydrolase L1*; *VPS35*, *vacuolar protein sorting 35*; *LRRK2*, *leucine-rich repeat kinase 2*; *Parkin*, *Parkin RBR E3 ubiquitin-protein ligase*; *PINK1*, *PTEN-induced kinase 1*; *GBA*, *glucocerebrosidase*; TCE, trichloroethylene; PERC, perchloroethylene; CCl_4_, carbon tetrachloride; Hg, mercury; Fe, iron; Cu, copper; Pb, lead; Mn, manganese; MPTP, 1-methyl-4-phenyl-1,2,3,6-tetrahydropyridine; MPP^+^, 1-methyl-4-phenylpyridinium ion; L-dopa, Levodopa; DA, dopamine; MAO-B, monoamine oxidase B; COMT, catechol-O-methyltransferase; LBs, Lewy bodies; UPS, ubiquitin-proteasome system.

In the year 1817, James Parkinson, a well renowned British physician, introduced the term PD, which was initially designated as paralysis agitans/shaking palsy [31,32]. PD is a debilitating, nerve-cell-deteriorating, and motor system ailment with multifactorial etiopathogenesis. It is represented by four principal motor manifestations, comprehending rigor/stiffness, bradykinesia/slowed movement, tremor/shaking, and postural impairment/abnormal body posture and balance (depicted in Figure 2) [5,6,33,34,35]. These manifestations make an appearance following the forfeiture of around 50–60% of DArgic nerve cells protruding through the SN-PC to the striatum and deprivation of nearly 80–85% of DA content within the striatum [36,37,38,39]. Owing to the substantial neuronal protection rendered by estrogen in women, it makes them considerably less susceptible to developing PD than men [8,9,10,11,12,13,40,41].

Being outlined by four classical manifestations, PD not only emerges through the escalation in the devastation and deprivation of DArgic nerve cells protruding through the SN-PC; instead, it is prompted via an amalgamation of genetic and environmental factors, as portrayed in Figure 2. In approximately 5–10% of individuals experiencing PD, the genetic profile serves a critical role, whereas the rest of the individuals experiencing PD appear to be of equivocal origins and, consequently, are regarded as idiopathic [42], with a little fraction strongly connected with mutations in genes, namely, *PARK1–18* [43]. Numerous investigations have elucidated that autosomal-dominant PD arises consequently to gene mutations that ciphers*α-synuclein*; a protein encoded by a gene named *PARK1* [44,45], *ubiquitin carboxy* (*C*)*-terminal hydrolase L1(UCHL1*); a biocatalyst enciphered by a gene named *UCLH1* [45,46], *leucine-rich repeat kinase 2* (*LRRK2*); and a biocatalyst ciphered by a gene named *LRRK2*/*PARK8* [45,47], and *vacuolar protein sorting 35* (*VPS35*) [48]. On the other hand, an autosomal non-dominant form of PD arises consequently to gene mutations, namely, *protein deglycase* (*DJ-1*), which is encoded by a gene named *PARK7* [49], *Parkin RBR E3 ubiquitin-protein ligase* (*Parkin*); encoded by a gene named *PARK2* [50], and *PTEN-induced kinase 1* (*PINK1*); and ciphered by gene named *PINK1* [51]. Apart from this, mutation in the *glucocerebrosidase* (*GBA*) gene also partakes in the evolution of PD [52,53].

Several studies have reported that exposure to environmental toxins, for instance, solvents (trichloroethylene (TCE) [54,55], carbon tetrachloride [55], and perchloroethylene (PERC) [55]), pesticides (paraquat [56], rotenone [56], and dieldrin [57]), fungicide (maneb) [58], mercury (Hg) [59], Fe [59], Cu [59], lead (Pb) [59], manganese (Mn) [59], and mitochondrial poison/neurotoxin1-methyl-4-phenyl-1,2,3,6-tetrahydropyridine (MPTP) [60], is strongly linked to an escalated susceptibility of PD expansion. The MPTP-instigated PD model appears to be one of the most important and beneficial models of such a debilitating malady in animals and cell cultures, which is distinguishable from human-associated PD in the context of etiology. It has been reported that MPTP undergoes bio-transformative conversion to an enormously active form, namely, 1-methyl-4-phenylpyridinium ion (MPP^+^), pertaining to nicotinamide adenine dinucleotide hydrogen (NADH)-ubiquinone oxidoreductase I suppressor category, and is actively engaged in eliciting the destruction of DArgic neurons pinpointed in the substantia nigra (SN) [61,62]. Owing to this, de-escalated operation of NADH-ubiquinone oxidoreductase I has been detected in thrombocytes and post-mortem examination of the brain in individuals experiencing sporadic forms of PD [63,64]. Numerous in vitro investigations have revealed that employment of agents, such as rotenone, and MPP^+^ might culminate in the emergence of oxidative damage, programmed cell death, as well as additional bio-transformative alterations, for instance, destruction of DNA, lipid peroxidation, and oxidation of protein (α-synuclein), which are also detected in individuals with idiopathic forms of PD [65,66]. Further, it has been elucidated that MPP^+^-provoked neuronal damage is facilitated via exorbitant nitric oxide (NO) generation through NO synthase (NOS) [67,68]. The NO formed can eventually experience interaction with superoxide (O_2_^−^), in order to produce a cytotoxic substance, namely, peroxynitrite (ONOO^−^), which in turn pursues irrevocable interaction with tyrosine so as to produce nitrotyrosine, which has been reported to alter the protein functioning and partake in the pathophysiology of a number of debilitating neurodegenerative conditions, encompassing PD [69,70].

The pathogenic events and evolution of PD are presumed to be robustly connected with significant features, for instance, aggregation of α-synucelin within the LBs in the greatly impacted regions of the brain [71]. The pathology of PD does not appear to be confined to the nigrostriatal region, since buildup of α-synucelin could be spotted around the entire nervous system, encompassing the cortex, locus coeruleus (LC), amygdala, enteric nervous system, peripheral autonomic system, sympathetic ganglia, pelvic and cardiac plexuses, adrenal medulla, skin, and submandibular gland [72,73,74,75,76]. These aggregates are principally composed of fibrillar, α-synucelin, parkin, synphilin, neurofilaments (NF), and neurotransmitter vesicle proteins. Furthermore, cognitive abnormality emerges following the frontal cortex and caudate nucleus degeneration [77], whereas non-motor manifestations associated with PD, such as sleep disruption, and olfactory and autonomic impairment, might arise approximately 10 years earlier from the emergence of motor manifestations [72,74,78]. Although the pathogenesis of the malady is elusive and abstruse, numerous hypotheses propose the significant involvement of multiple pathways in the worsening and advancement of the disease (as illustrated in Figure 2) [15,16,17,34].

At present, existing drug therapy for PD (as depicted in Figure 2) can only furnish incomplete alleviation of the manifestations, and exhibits no effect on the evolution of the malady [18,19]. The leading pharmacotherapy comprehends a precursor of DA termed L-dopa, which may permeate through the blood–brain barrier (BBB) to undergo transformation into DA through DArgic nerve cells by means of an enzyme named DA decarboxylase [79,80]. In order to facilitate the diffusion of L-dopa through the BBB to pursue the nerve cell-mediated transformation to DA, and to preclude systemic biotransformation, L-dopa is generally introduced with a DA decarboxylase suppressor. The occurrence of multifarious therapy-associated consequences, such as dyskinesia, variation in therapeutic outcomes, and the emergence of psychological conditions, may ultimately impede the utilization of L-dopa in medical practice [81]. On the other hand, agonists of DA can be employed to cure such a disabling condition. Employment of agents pertaining to the class of monoamine oxidase B (MAO-B) and catechol-O-methyltransferase (COMT) blockers can successfully regulate DA deprivation in the brain [82]. Aside from that, non-steroidal anti-inflammatory drugs (NSAIDs) (aspirin and ibuprofen) [83], leukotriene receptor antagonist (montelukast) [84], vitamins (B_3_, D, E, and C) [85], alcohol imbibing [86], smoking [86], caffeine intake [87], and physical exercise [88] have been demonstrated to render significant neuronal protection in PD. Recent investigation has revealed that escalated alcohol consumption possesses safeguarding effect over the risk of PD (with odds ratio 0.79; 95% confidence interval 0.65–0.96; probability value = 0.021), with the significant involvement of *alcohol dehydrogenase 1B* [86]. Likewise, continuation of smoking (only while involving the comparison between current and former smokers) (with odds ratio 0.64; 95% confidence interval 0.46–0.89; probability value = 0.008) has been revealed to exhibit safeguarding outcomes in PD [86]. Another meta-analysis has explored the association between consumption of alcohol and the progression of PD. According to this meta-analysis encompassing 32 studies and involving nearly 677,550 subjects, it has been revealed that only beer (with risk ratio = 0.59; 95% confidence intervals: 0.39–0.90), and not liquor/wine, protected against the PD emanation [89,90]. In addition, a massive meta-analysis encompassing 8 cohort and 44 case-referent studies from different regions of the world described an inverse association between smoking and PD (with a total risk of 0.39 for current smokers) [91]. Another study has also reported an inverse association between the total amount of pack years, years of smoking, and the risk of PD, with persistent smokers exhibiting a comparably de-escalated risk of developing PD than non-smokers [92]. However, the exact constituents/mechanisms underlying the safeguarding effects of alcohol consumption and smoking in PD remains unclear. Therefore, further investigation is wanted to elucidate the constituents/mechanisms implicated behind the alcohol and smoking mediated neuronal protection, and to attain evidence-based results. Lastly, considering the irrevocable evolution, as well as the intricate and diverse nature, of the condition, the immense unfulfilled pharmacological requirement is the recognition of appropriate and efficient pharmacotherapy possessing disease-modifying and nerve-cell-protective abilities [19]. Although a number of therapies are being discovered, more exploration is highly required in order to entirely recognize the pivotal pathogenic mechanisms, which upon selective targeting might aid in the development of pharmacotherapy, which assists in the complete cessation of the malady.

## 3. Oxidative Stress: An Imperative Player in PD

It has been promulgated that significant escalation in ROS concentrations beyond the cell’s antioxidant abilities culminates in making the cells immensely susceptible to OS, which in turn prompts irrevocable devastation of cells and finally contributes to cellular demise. ROS might be generated in an inordinate manner, for instance, hydrogen peroxide (H_2_O_2_), O_2_^−^, NO, singlet O_2_, and ONOO^−^ [93]. Profusion in the ROS generation might culminate in lipid peroxidation, forfeiture of unification of DNA, protein (α-synuclein) misfolding, and abnormalities in the mitochondrial operation that may deteriorate the nerve cells [94]. Numerous antioxidants, namely, superoxide dismutase (SOD), vitamins (E, C, and D), catalase (CAT), and glutathione (GSH) peroxidase, are revealed to be actively engaged in de-escalating the pernicious ROS to such concentrations that are non-pernicious for the body [85,95]. The human brain is presumed to be one of the tremendously prone regions to pernicious ROS and oxidative destruction in comparison to other regions of the body. The human brain utilizes nearly 18–20% of the O_2_ available in the physiological system, with a preponderance proportion of the O_2_ being transformed to ROS owing to the extortionate O_2_ biotransformation of nerve cells [96,97]. Additionally, in order to tackle ROS, the human brain displays inadequate quantities of biocatalysts possessing free-radical scavenging activity and a remarkably limited antioxidant network [98,99,100]. Moreover, the nerve cells’ proneness towards oxidative destruction, which principally builds up in nerve cells experiencing ageing, may also be owing to the diminished ability of cells to undergo mitosis [101]. In the presence of OS, extortionate levels of polyunsaturated fatty acids (PUFAs) of a promptly oxidizing character inside the brain are extremely vulnerable to lipid peroxidation and pernicious radical constituent formation. Consequently, OS emerges as a key player that partakes in the commencement and evolution of nerve-cell-deteriorating maladies.

Parkinson’s disease, an intricate, elaborative, and multifaceted nerve-cell-deteriorating disease, is marked by the deprivation of the DArgic nerve cells of the nigrostriatal region [34]. Present-day scientific corroboration indicates that OS critically partakes in the DArgic programmed cell death in PD [102,103,104]. To illustrate, a pivotal oxidant, namely, H_2_O_2_, is produced following the emergence of OS and might culminate in the programmed cell death of nerve cells [105]. Further, a pesticide, namely, rotenone, which has been elucidated to exhibit pernicious repercussions on nerve cells of experimental models with PD, may give rise to the escalated H_2_O_2_ generation, and as a consequence contribute to the programmed cell death of nerve cells [106,107]. Moreover, post-mortem examination of the brains of individuals experiencing PD validates the active participation of OS in the emanation of the ailment. This type of corroboration for the engagement of OS in the brains of individuals experiencing PD encompasses an escalated unbound Fe concentration, oxidation of DNA, augmented operation of SOD, substantial forfeit of GSH, and de-escalation in the operation of mitochondrial complex I [108,109]. In addition, several biological markers associated with OS are tremendously elevated in systemic circulation and cerebrospinal fluid (CSF) (such as the generation of O_2_^−^, and levels of malondialdehyde (MDA)) of individuals experiencing PD [110,111]. Even though recent investigations have reported that escalated OS as a result elevates the predisposition of α-synuclein to build up within the cells, whether these alterations in α-synuclein are pernicious or safeguarding to the nerve cells continues to remain ambiguous [112,113,114]. The production of pernicious ROS and oxidative devastation are presumed to be actively engaged in the DArgic nerve cells demise [115]. Redox-active metals, for instance, Fe and Cu, being imperative ROS generators, have been revealed to be accrued in the brains of individuals experiencing PD [116]. The current corroborations clearly demonstrate that redox-active metals may have a significant contribution in the evolution of PD through ROS-reliant mechanisms.

## 4. Metabolism of Iron in the CNS

**Figure 3:**
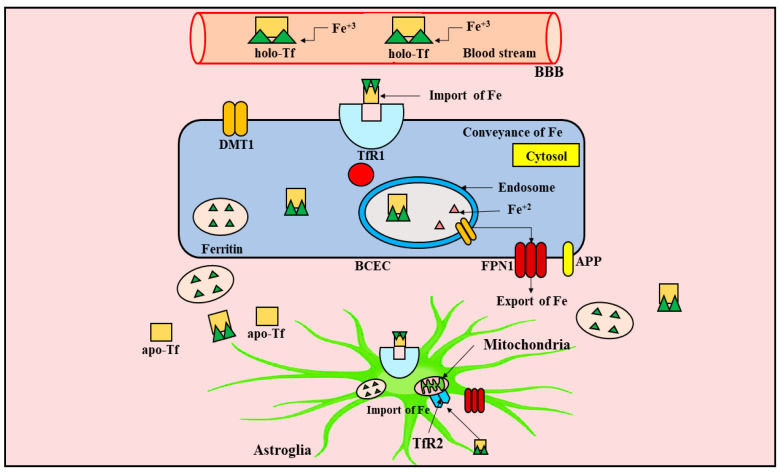
Conveyance and metabolism of Iron. The preponderance of Fe present within the blood stream is attached to Tf. The conveyance of Fe to the brain is provoked via Tf that undergoes interaction with TfR1 pinpointed exterior to the BCECs. The complex formed, namely, Tf-TfR1, is engulfed within the endosome via endocytosis. Within the endosomes, Fe experiences dissociation and transformation from Fe^+3^ to Fe^+2^ via a specific metalloreductase. The principal transporter of Fe, namely, DMT1, elicits the conveyance of Fe from endosomes to the fluid present within the living cells (cytosol), whereupon it encounters re-joining with Tf. Further, the mitochondria uptakes Fe for performing cardinal operations, such as the regulation of ETC functioning, and the generation of Fe-S clusters and haem via TfR2. FPN1 exists as the primary exporter of Fe present inside the cell. The ferroxidases carry out the oxidation of Fe following its exportation. In addition, APP promotes the exportation of Fe by interacting with FPN1. Fe, iron; Tf, tarnsferrin; Fe^+3^, ferric Fe; holo-Tf, holo-transferrin; BBB, blood–brain barrier; TfR1, Tf receptor 1; DMT1, divalent metal transporter 1; Fe^+2^, ferrous Fe; FPN1, ferroportin 1; APP, amyloid precursor protein; BCEC, brain capillary endothelial cell; apo-Tf, apo-transferrin; TfR2, Tf receptor 2; ETC, electron transport chain; S, sulphur.

Iron is presumed to be an extremely important element in the bio-transformative reactions occurring within the mammalian body, owing to its significant contribution in the synthesis of Fe-sulphur (S) clusters, heme, and also serves as a cofactor in several other bio-transformative processes. In the absence of Fe, red blood cells (RBCs) lose their ability to carry out the conveyance of haemoglobin (Hb)-attached O_2_ to the body tissues, and oxidative phosphorylation by way of electron transport chain (ETC) complex, which encompasses 12 Fe-S groups, and 7 haems, would be unattainable [117]. Extensive knowledge regarding the metabolism of Fe within the CNS has become extremely critical, since scientific data strongly propounds the implication of brain Fe metabolism during progression of numerous nerve-cell-deteriorating ailments, for instance, PD, ALS, and AD. The conveyance and metabolism of Fe is depicted in Figure 3. It has been reported that the interaction of blood-plasma glycoprotein named transferrin (Tf) with the Tf receptor 1 (TfR1) pinpointed superficially upon brain capillary endothelial cells (BCECs) brings about uptake of Fe through the nerve cells. The complex formed between Tf and TfR1, i.e., Tf-TfR1 is incorporated within membrane-attached vesicles termed endosomes via a process named endocytosis, whereupon Fe dissociates from Tf, owing to the decline in potential hydrogen (pH). Within endosomes, a metalloreductase termed six-transmembrane epithelial antigen of prostate 3 (STEAP3) converts Fe from Fe^+3^ to Fe^+2^ state [118], and is subsequently conveyed across the endosomal membrane to the fluid present within the living cells, termed cytosol, through the prime Fe transporter termed divalent metal transporter 1 (DMT1) [119].

Following its conveyance inside the cytoplasm, Fe may be further employed for nerve cell operation and biotransformation, whereas the exorbitant Fe experiences complexation with ferritin and gets deposited or eliminated from the nerve cells via ferroportin 1 (FPN1) and oxidized through moving or astrocyte-attached ceruloplasmin (CP) before being loaded to Tf located outside the cell [120].

Further, the two ZRT/IRT-like proteins (ZIP), namely, ZIP8, and ZIP14, pertaining to the class of ZIP solute carriers, can straightforwardly carry the conveyance of non-Tf bound Fe (NTBI) throughout the biological membrane [121,122,123]. ZIP14, analogous to DMT1, possesses the ability to carry out the conveyance of Tf-attached Fe from the endosome to the cytoplasmic region [123]. Both ZIP8 and ZIP14 appear to be neoteric Fe transporters which might exhibit different physiological activities. As an example, the Fe conveying capability of ZIP8/ZIP14 is maximum beyond neutral pH (pH 7), whereas DMT1 works most appropriately at weakly acidic pH (pH 5.5) [121,122,124]. Furthermore, ZIP8 seems to be a chief conveyer of NTBI within rat hippocampal nerve cells, contrarily to DMT1, which principally conveys Tf-attached Fe [125]. Additionally, several studies have revealed that a Fe chaperone protein termed poly r(C)-binding protein (PCBP) holds the aptitude to convey Fe in the direction of ferritin to pursue efficient buildup, as well as to the non-haem Fe demanding proteins, namely, deoxy hypusine hydroxylase (DOHH), and hypoxia-inducible factor (HIF)-prolyl hydroxylase (PH) and asparaginyl hydroxylase [126,127,128].

It has been elucidated that mammalian proteins, namely, Fe regulatory proteins (IRPs), further bifurcated as IRP1 and IRP2, and are actively engaged in the modulation of expression of genes implicated in the metabolism of Fe, in order to retain stable concentrations of Fe within the cell. IRPs interact with Fe-responsive elements (IREs) pinpointed on the messenger ribonucleic acid (mRNA) transcripts, thereby escalating the import of Fe, and diminishing employment, storage, and export of Fe. Being RNA-interacting proteins, the two, IRP1 and IRP2, have the potential to change the TfR mRNA, FPN, and ferritin translation [129,130]. Further, Fe present within the cytosol might interact with IRPs, prompting a structural alteration that restrains association with IREs pattern within mRNA. On the other hand, a significant de-escalation in the levels of Fe permits IRPs to frequently interact with IREs, so as to alter translation. Besides, the protein transcripts, for instance, FPN, and ferritin, which have been revealed to partake in storing/exporting Fe, safeguard IRE pinpointed on the small chain of intramolecularly attached RNA/DNA (stem-loop) configurations of the 5′-untranslated region(5′-UTR), and IRP interaction culminates in the preclusion of the translation process and facilitation of breakdown. In contrast, Fe importing protein transcripts like DMT1, and TfR1, have been demonstrated to safeguard IRE on their 3′-UTR, and consequently are translated and maintained in the course of Fe deprivation. In order to impede the generation of ROS and oxidative destruction, Fe needs to be buffered and stored in an appropriate manner. It has been promulgated that ferritin constitutes the fundamental Fe-storing protein in nerve cells and non-nerve cells (neuroglia/glial cells), whereas an intricate dark pigment detected in the brain, namely, neuromelanin (NM), ensnares enormous quantities of Fe with the ambition of storing it for prolonged duration of time, particularly in neuronal networks, namely, DArgic nerve cells of the SN region of the brain [131]. Furthermore, Fe can be liberated and utilized again from ferritin via ferritinophagy, a process involving the autophagy-lysosomal mediated destruction of ferritin [132]. Moreover, an enzyme named heme oxygenase-1 (HO-1) might prompt heme breakdown to Fe^+2^ as a means of maintaining the balance of Fe within the body [133].

## 5. Metabolism of Copper in the CNS

**Figure 4:**
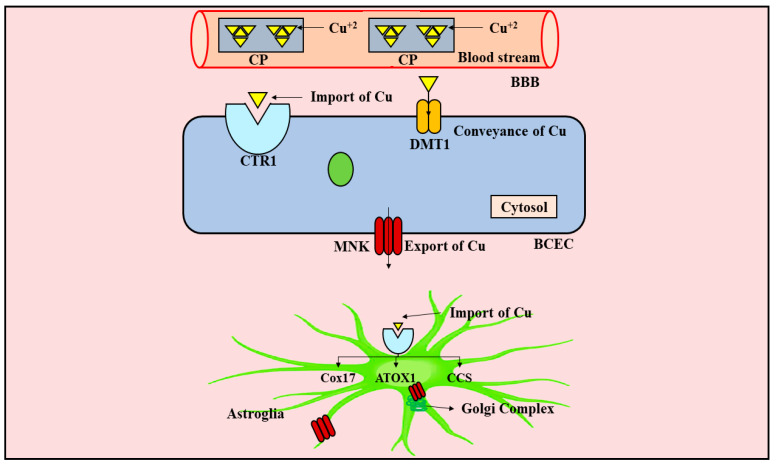
Conveyance and metabolism of copper. The preponderance of Cu present within the blood stream is attached to CP. The CTR1 pinpointed exterior to the BCECs is actively engaged in the uptake of Cu inside the brain. Further, the membrane proteins, namely, P-type ATPases, are the protein comprising conveyers that react to surges in the levels of Cu inside the cell by trafficking to the membrane and thereby facilitating the export of Cu. Once inside the cytosol, Cu interacts with chaperones, namely, Cox17, ATOX1, and CCS, that assist in the conveyance of Cu to their respective cupro-biological molecules. Cu, copper; Cu^+2^, cupric form of Cu; CP, ceruloplasmin; BBB, blood–brain barrier; CTR1, Cu-transporter 1; DMT1, divalent metal transporter 1; MNK, Menkes protein; BCEC, brain capillary endothelial cell; Cox17, Cu chaperone for cytochrome c oxidase; ATOX1, antioxidant 1 Cu chaperone; CCS, Cu chaperone for SOD1; SOD1, superoxide dismutase 1; ATPases, adenosine triphosphate-ases.

Copper is considered to be an eminently vital element that is actively engaged in the appropriate operation of key biocatalysts. However, owing to the redox-active nature of Cu, it may significantly contribute to OS via escalated production of deleterious ROS [134]. Consequently, the uptake, storage, and exportation of Cu inside the cell should be adequately controlled so as to ensure optimal availability of Cu to bring about the production of biocatalysts comprising Cu, and simultaneously to restrain oxidative damage provoked by the presence of Cu. Cu is critically needed for the optimal operation of the brain. Furthermore, both profusion and scarcity of Cu might have detrimental repercussions on the functioning of the brain. As a result, the brain exhibits multifarious pathways for modulating the metabolism of Cu. Astroglia have been presumed to be integral modulators of Cu equilibrium within the brain [134]. Dys-equilibrium in Cu biotransformation within the CNS is strongly linked with numerous neurodegenerative conditions, including PD, AD, and ALS, and, therefore, in-depth understanding of Cu metabolism in the CNS is of great importance nowadays. The conveyance and metabolism of Cu is depicted in Figure 4.

It has been revealed that Cu is predominantly attached to CP within the plasma, and a little fraction is attached to a Cu carrier (transcuperin), amino acids (AA), and a protein (albumin) [135,136]. Cu is principally conveyed to the brain in the free ionic form via two routes, namely, the BBB (greater proportion), and the blood CSF barrier (little proportion) [135]. Cu-transporter 1 (CTR1) is primarily accountable for bringing about Cu uptake inside the nerve cells, and once within the cytosol it undergoes interaction with chaperones, namely, Cu chaperone for cytochrome c oxidase (Cox17), antioxidant 1 Cu chaperone (ATOX1), and Cu chaperone for SOD1 (CCS), which partake in the effective conveyance of Cu to particular biocatalysts. In order to preclude the redox-active nature of Cu, and the generation of ROS, the surfeit of Cu experiences chelation (complex formation) with an antioxidant named GSH (in millimolar concentration) [137], and proteins named metallothioneins (MTs) (in millimolar concentration) [138]. The membrane proteins, namely, P-type adenosine triphosphate (ATP)-ases, viz., ATPase Cu-transporting alpha (ATP7A)/Menkes protein (MNK), and ATPase Cu-transporting beta (ATP7B)/Wilson disease protein (WND), are most often pinpointed in the Golgi complex membrane, whereupon they culminate in the loading of Cu onto enzymes termed cuproenzymes, such as DA β-hydroxylase, and CP [139,140]. Following an escalation in the levels of Cu inside the cell, ATP7A carries out the conveyance of Cu across the cell membrane [141,142]. Further, translocation of Cu from ATOX1 to MNK or WND culminates in the elevated functioning of ATPase, whereas ATOX1 possesses the ability to recover Cu from such ATPases and result in their diminished functioning when Cu is available in its unbound state.

## 6. Implication and Disrupted Balance of Redox-Active Metals in PD

### 6.1. Implication of Iron in PD

Iron is regarded as a pivotal element actively engaged in integral physiological operations, for instance, synthesis of DNA, mitochondrial respiration, and conveyance of O_2_ [120]. Additionally, Fe also partakes in the modulation of the generation of safeguarding envelops that enclose axons (myelin sheath), neurotransmitters, as well as the extension and recreation of nerve cell projections (dendritic spines) within the hippocampal region of the brain [143]. It has been revealed that the human brain exhibits a varied distribution of Fe, with smaller proportions in the cortical white matter, gray matter, cerebellum, and mesencephalon, and larger proportions in the basal nuclei (caudate, putamen, and dorsal pallidum) [120]. An infinitesimal change in the proportion of Fe culminates in the diminished performance of the brain, such as cognitive abnormality, and lack of myelination of the nerve cells [144]. Owing to the fact that Fe serves as a cofactor for tyrosine 3-monooxygenase, which impedes neurotransmitter production and partakes in the deprivation of DA, the Fe buildup within the SN region of the brain gives rise to deleterious consequences in PD [145]. Consequently, the abundance and scantiness of metals of a redox-active nature, especially Fe and Cu, might be detrimental to DArgic nerve cells. Despite the fact that Fe is enormously crucial for biological operations in numerous regions of the body, comprehending the brain, several corroborations illustrating the pernicious effects of Fe on nerve cells has been postulated [146,147,148], particularly on tyrosine hydroxylase (TH)-positive nerve cells [148]. Numerous experimental findings have elucidated that substantial escalation in the Fe levels was observed during ultrasound and magnetic resonance imaging (MRI) testing inside the melanised DArgic nerve cells of individuals experiencing PD [149,150,151,152,153,154,155,156]. Furthermore, it has been reported that granules of NM with profuse Fe were detected near the nerve cells inside the SN of individuals experiencing PD [157]. An upsurge in the concentrations of Fe was also recognized within the SN of various experimental models with PD, for instance, rotenone [158], MPTP [159], 6-hydroxy DA (6-OHDA) [159], and lactacystin [160]. Recent investigations have elucidated that inordinate overloading of Fe might culminate in the escalated intracellular Ca ion (Ca^2+^)-reliant stimulation of protein phosphatase 2B (PP2B)/calcineurin (CaN) inside the nerve cells pinpointed in the hippocampal region, elevated activation of glutamate receptors resulting in raised excitotoxicity, and eventually contributes to nerve cell deterioration. Following subjection to escalated levels of Fe, human neuroblastoma (SH-SY5Y) DArgic nerve cells experience persistent buildup of Fe, culminating in a two-phase shift wherein low levels of Fe uplift the concentration of an antioxidant, namely, GSH, which declines afterwards [161].

Furthermore, existing publications have revealed that inordinate Fe buildup in the SN-PC and lateral dorsal pallidum precipitates the DArgic nerve cell deprivation through immensely reactive hydroxyl radicals (·OH) or ROS generation via the Haber–Weiss and Fenton reaction, which give rise to impaired conformational unification of nerve cells [162,163,164]. In PD, the ROS generation, in amalgamation with forfeit of antioxidants, primarily GSH, can contribute to OS [165]. In addition, Fe partakes in the lipid peroxide degradation, culminating in the formation of free radicals that are immensely deleterious in nature and might exhibit devastating effects on DNA, proteins, and lipids, consequently contributing to the demise of nerve cells in experimental models with PD [165].

Apart from this, Fe’s participation in PD is greatly connected to its aptitude to relate oxidative devastation and buildup of α-synuclein. Several investigations have delineated that the buildup of Fe inside the LBs and the lipid peroxidation provoked by Fe culminates in the significant enhancement of the buildup of α-synuclein [165]. According to in vitro examinations, Fe precipitates the oxidation within the cells and additional α-synuclein buildup, along with mitochondrial aggregation, inside the DArgic nerve cells [166,167,168,169]. Other experiments have indicated that Fe modulates the buildup of α-synuclein inside the SK-N-SH neuroblastoma cells via the IRE or IRP pathway [168], and OS greatly impacts the buildup of α-synuclein through oxidizing Fe to the Fe^+3^ form [170]. In addition, within the SN-PC of individuals experiencing PD, Fe facilitates the transformation of α-helix α-synuclein to β-pleated sheet α-synuclein [171]. α-synuclein is an extremely acidic protein enciphered by a gene, namely, *SNCA*, that exhibits a C-terminus encompassing around 38% (16–42) acidic AA, namely, aspartate, and glutamate. The duo aspartate and glutamate establish the conditions optimal for the interaction of Fe in the way ferritin interacts with its protein named NF. At micromolar concentrations, α-synuclein experiences association with Fe^+2^ inside the cell and culminates in the generation of α-synuclein aggregates, relying upon dosing pattern. In the course of LB malady and ageing, the unbound Fe buildup significantly elevated the α-synuclein clumping/aggregation within the SN [172].

Another investigation has promulgated that MPP^+^ in a dose of nearly 0.25–0.50 μm is capable of eliciting the implosion of neuritic tree inside the mesencephalic nerve cells, with no substantial forfeit of cellular operability [173]. This MPP^+^-effectuated effect might be precluded by de-escalating the intake of Fe or by supplementing with agents having an antioxidant nature. As a consequence, it is presumed that escalated ROS and Fe levels inside the cells critically partake in the commencing phases of DArgic nerve cell impairment before cellular demise. Afterwards, a continuous loop encompassing the buildup of Fe, impairment in complex I operation, and escalation in the quantities of ROS might contribute to irrevocable oxidative destruction and nerve cell death.

Moreover, the SN-PC gives the impression of being enormously susceptible to OS, owing to its innately escalated levels of Fe and DA synthesis [174,175]. The pernicious association between Fe and DA is the noteworthy pathway via which Fe partakes in the progression of the ailment. DA pertains to the class of catecholamine (CA) and is the fundamental neurotransmitter pinpointed inside the human brain, and its conveyance via DArgic nerve cells is actively engaged in controlling cognitive, reward, and motor operations. Regardless of its fundamental conveying operation, it is profoundly prone to undergoing oxidation, which in turn culminates in the generation and simultaneous liberation of species that may be pernicious for the nerve cells [174]. The duo, namely, Fe^+3^ and Cu^+2^, hold the potential to bring about DA oxidation during the existence of O_2_. Due to the buildup of mobile and fluctuating Fe in the SN-PC, it potentially partakes in DA oxidation. Secondary species of these reactions comprehend DA quinone (DAQ), and DA semiquinone radical (DASQ), which may exert pernicious consequences along with H_2_O_2_, O_2_^−^, and OH, might be generated as a result of elaborative reactions [176]. The aforementioned products may elicit OS and impairment in mitochondrial operation, as well as prompt and balance the generation of oligomeric α-synuclein [177,178]. Apart from this, Fe and Cu can also facilitate the biocatalyst-mediated deamination of DA via MAO, which culminates in the formation of H_2_O_2_, and an acidic metabolite of DA, namely, 3,4-dihydroxyphenylacetic acid (DOPAC), which in turn can contribute to additional ROS formation [179]. Consequently, in a condition that favors OS (namely, buildup of Fe and Cu) and oxidation of DA, it might be deleterious for the nerve cells, inducing destruction of synaptic boutons and neurotransmitter-comprising vesicles.

In view of the fact that the buildup of Fe in the impacted regions is an imperative circumstance in PD, metal reduction (with the aid of Fe chelators, namely, desferrioxamine (DFO), deferiprone (DFP), and deferasirox (DFX)) could be a neoteric and futuristic treatment approach for PD [180,181,182,183,184] and is described in detail in the following sections.

### 6.2. Implication of Copper in PD

Copper is regarded as an imperative element needed for the operation of biocatalysts that are actively engaged in the synthesis of neurotransmitters, and the generation of energy for the appropriate functioning of the mitochondria. It has been elucidated that Cu minimizes OS via acting as a cofactor for prime biocatalysts exhibiting anti-oxidant abilities, namely, Cu/Zn SOD [185]. Cu, being a metallic biomaterial, is crucial for the optimal operations of the human brain, for instance, modulation of conveyance of nerve cells, γ-aminobutyric acid A (GABA_A_), and N-methyl D-aspartate (NMDA) receptors [186]. Further, the unbound Cu holds the aptitude to elevate OS, the formation of LBs, and the oligomerization of α-synuclein via two reactions, namely, the Fenton reaction and the Haber–Weiss reaction. Another investigation has elucidated that persistent subjection to Cu for nearly two decades escalated the incidence of PD [165]. Additional investigations on individuals residing in urban regions have demonstrated that PD occurrence rate is higher in regions emitting considerable levels of Cu/Mn [187]. Moreover, Cu content was found to be escalated throughout the DArgic networks in an experimental model with PD employing 6-OHDA [188]. Consequently, Cu profusion might significantly partake in the commencement or evolution of PD.

The profound ROS formation is frequent in the case of PD, as corroborated by an elevation in the lipid peroxidation levels in the blood samples of individuals experiencing the malady. Cu ions are presumed to be actively engaged in this pathway [165]. To give an instance, subjection of rats to Cu (for nearly 28 days) in the water fed to them for drinking purposes exhibited de-escalated operation of SOD in their brains and elevated contents of the marker of lipid oxidation, namely, MDA [189]. Further, it has been revealed that the straightforward introduction of Cu sulphate (CuSO_4_) within the rodent SN might result in deleterious effects on DArgic nerve cells, comprehending de-escalated DA, escalated OS, and programmed cell death [190]. Escalation in the Cu content outside the cell culminates in nerve cell death via enhancing the pernicious ROS formation [165]. Cu, in particular, greatly impacts the liberation of molecules that partake in safeguarding the nerve cells from OS, for instance, cyclophilin A (CyPA), and mediators of inflammation, for instance, interleukin-1 (IL-1) [191]. Therefore, it can be said that pernicious repercussions of Cu on the nerve cells may be prompted via the ROS-reliant pathway. However, it is ambiguous whether ROS or Cu is the initiator of DArgic nerve cell deterioration. Even though inordinate Cu levels can culminate in nerve cell deterioration, scarcity in the absorption of Cu might also exhibit numerous pernicious effects, for instance, intercedence in the operation of Cu-comprising biocatalysts contributing to the impaired interaction of proteins in the matrix pinpointed outside the cells and variations in cellular conveyance [192]. The pernicious repercussions of diminished Cu content might be attributed to the de-escalated operation of a biocatalyst named SOD. As a result, maintaining a balance in Cu levels within the physiological system is critical.

Furthermore, it has been reported that at optimal biological pH, the interacting position for Cu^+2^ is existent, analogous to the high-affinity N-terminal M1-D2 binding site and the low-affinity H50 binding site also pinpointed at the N-terminus [185,193,194]. However, nearly at weakly acidic pH, i.e., pH 5, the H50 interaction is significantly de-escalated, and α-synuclein also exhibited a somewhat distinct interacting site for Cu at D119-E123 [185]. Interestingly, the participation of Cu in the pathogenic events implicated in PD is also presumed to be related to its aptitude to interact with α-synuclein to create a complex with it. The buildup of α-synuclein is postulated to be an imperative episode actively engaged in the evolution of PD [195]. Numerous investigations have demonstrated that Cu partakes in the promotion of α-synuclein buildup [196,197]. Moreover, it has been reported that Cu plays a pivotal role in the α-synuclein buildup, and de-escalation in Cu content inside the cells in turn contributes to diminished buildup generation [198]. This study has also revealed that de-escalation in Cu content provokes alteration in α-synuclein positioning, with reduced Cu content making it tremendously positioned towards the cell membrane, and this alteration is reverted following the reintroduction of Cu within the cells [198]. Another investigation has indicated that via regulating the breakdown of protein, inordinate expression of α-synuclein at non-pernicious concentrations escalates the DArgic cell demise prompted by subjection to Cu [199]. Moreover, the pivotal proteins implicated in the modulation of Cu, namely, MTs, pinpointed within the mammalian cells, tremendously influence the buildup of α-synuclein in a Cu-reliant way. Further, the α-synuclein interacts with Cu, potentially allowing additional Fe to be available for free radical production [200]. Cu is also actively engaged in the modulation of Fe levels inside the brain via the operation of a liver-generated enzyme and ferroxidase, namely, CP. Escalated levels of Fe in individuals experiencing PD aid the substantially reduced Cu and CP levels inside the human brain. Escalated amounts of unbound Cu, on the other hand, can contribute to the forfeiture of ferroxidase operation inside the CSF. Furthermore, it has been reported that Fe buildup within the human brain is profoundly linked to such an incapacitating neurodegenerative malady. Howbeit, the way by which a minute proportion of the Cu/Cu protein precipitates the disruption in the Fe balance in PD remains abstruse [201].

Existing treatment approaches, for instance, the introduction of L-dopa, DA agonists, and antioxidants, can only render incomplete alleviation of manifestations associated with PD. As a result, formulating a suitable Cu chelator in order to switch from manifestation-alleviating to malady-altering/terminating pharmacotherapy remains critical.

### 6.3. Disrupted Balance of Iron and Copper in PD

Discrepancies exist in the published literature examining the changes in the levels of Fe in individuals experiencing PD. Several studies have elucidated that the levels of Fe were found to be tremendously escalated in individuals experiencing PD, and postmortem examination of neural tissue, particularly in the DArgic nerve cells protruding through SN-PC [154,202,203,204,205,206]. On the other hand, a recent investigation focusing on examining the amounts of nine critical metals (Fe, Cu, Mn, Zn, selenium, Mg, K, Na, and Ca) in nine different areas of the brain (primary motor cortex, cingulate gyrus, primary visual cortex, hippocampus, cerebral cortex, SN, LC, medulla oblongata, and middle temporal gyrus) in individuals experiencing PD dementia has revealed no alterations or changes in the levels of Fe inside the SN or other detected areas of the brain [207]. Another study has demonstrated that de-escalated levels of Fe, plausibly owing to the increased energy demand during the initial stages of PD (as displayed by MPTP-subjected monkeys, namely, Mk1 and Mk2), significantly contributed to the early stage of the PD [208]. During the commencement of PD, the loss of nerve cells is profoundly confined to the SN-PC [209]. A wide range of corroboration strongly suggests that numerous factors in conjunction are immensely associated with the buildup of Fe within the brain of individuals experiencing PD. These comprehend DMT1 up-regulation in the DA-containing nerve cells [210], elevated expression of inflammatory mediators [211], raised BBB penetration or impairment [212], escalated receptors for lactoferrin (Lf) within the nerve cells and micro vessels [213], and mutations/alterations in genes strongly associated with the interaction and conveyance of Fe [214]. Furthermore, the levels of ferritin within the SN were found to be de-escalated, which might be owing to the prolonged operation of IRP1 detected during the postmortem investigation of the brain of PD patients, and which could significantly influence the deposition/storage of Fe [215]. In addition, down-regulation of FPN is recognized in numerous nerve cell toxin-reliant experimental models of mice experiencing PD, such as 6-OHDA, and MPTP [216]. The loading of Fe is marked by considerable elevation in Fe levels, and de-escalation in the levels of ferritin, which eventually culminates in a condition highly vulnerable to oxidative destruction.

Contrarily, several studies indicate substantially greater Cu levels within the CSF, particularly in individuals experiencing PD, presumably due to the alterations in the structure of proteins, and OS [217,218]. Moreover, the levels of Cu were also found to be considerably high in blood serum of individuals experiencing PD, which, as a result, is directly related to the grievousness of the malady [219]. In contrast, both the levels of Cu, as well as Cu attached to NM, within the deteriorating areas and the SN of PD patients were found to be de-escalated [216,220]. Abnormality in the conveyance of Cu in PD might be accountable for the de-escalated levels of Cu in the SN. Besides, individuals experiencing PD display a significant decline in CTR1 expression in the SN [186,220,221]. SOD1 experiences a lack of metallation in PD, owing to the diminished Cu content [219]. This could greatly impact the operation of SOD1, which is considered necessary for the eradication of ROS. Further, the proteins, namely, MTs, provoke the deposition/buildup and sequestration of Cu, thereby restraining the OS. Besides, MT3, an isotype particularly pinpointed in the human brain, was found to be diminished in PD, which might be linked with the elevated proneness to OS [222].

## 7. Implication of Metal Complexes and Metal Chelators in the Therapy of PD

For the purpose of investigating the pathogenic events implicated in the PD evolution, and establishing neoteric and propitious treatment targets, neurotoxin experimental mouse models have been eminently employed. The neurotoxins, namely, 6-OHDA and MPTP, notably focus on targeting and carrying out the forfeiture/devastation of DArgic nerve cells [223,224]. After the introduction of the aforenamed neurotoxins, the number of DA-generating nerve cells in the SN-PC drops by about half, and mice experience motor abnormalities analogous to those spotted in human beings during delayed phases of PD. It has been revealed that Cu complexed diacetyl-bis(4-methylthiosemicarbazonato) Cu^II^ (Cu^II^atsm) [225], and numerous Fe chelators, such as DFO [226], DFP [226], and DFX [226], have been examined as promising therapies employing these models.

The Cu^II^atsm, a Cu complex, has been extensively explored as a promising therapy for neurodegenerative ailments, such as PD and ALS. Cu^II^atsm exists as a low molar weight (around 321.9) and thermodynamically stable complex with lipid-loving/BBB-permeable and neutral attributes, possessing numerous therapeutic applications, mainly as an anti-neoplastic agent [227,228,229]. Cu^II^atsm has recently been employed as a drug for carrying out imaging scans, namely, positron emission tomography (PET), so as to designate regions deprived of the O_2_ supply within the peripheral tissues and the CNS [230,231,232,233]. Owing to its high stability and minimal deleterious consequences, it is widely recognized as an excellent pharmacological agent [228]. Further, Cu^II^atsm introduction precipitates the O_2_-deprived tissue retention owing to abnormalities in the mitochondria within the ETC, and has been outlined in PD. Additionally, it has been elucidated that Cu^II^atsm builds up in the striatal region, and escalation in the buildup was detected with the advancement in the ailment [233]. Up to the present time, Cu^II^atsm is investigated in numerous mouse models experiencing PD, and each of them has demonstrated propitious effects, encompassing enhanced motor and cognitive operations, upgraded biotransformation of DA, and restoration of DA-producing nerve cell destruction in the SN-PC [234]. It has been delineated that Cu^II^atsm operates via scavenging ONOO^−^, suppressing the emergence of associated deleterious consequences, and precluding nitro (NO_2_) group addition (nitration) and interaction between polypeptide chains (oligomerization) of α-synuclein [234]. Furthermore, therapy with the aid of Cu^II^atsm might culminate in escalated functioning of the biocatalyst, namely, TH, which is actively engaged in eliciting the l-tyrosine hydroxylation in order to generate the DA precursor, namely, L-dopa. This investigation has demonstrated a twofold escalation in the DA concentration following therapy with the assistance of Cu^II^atsm, but does not display a return to the optimal control ranges [234]. Howbeit, the escalated concentrations of DA were enough to remarkably upgrade motor operations. Apart from this, Cu^II^atsm holds the aptitude to up-regulate the expression of a critical protein, namely, vesicular monoamine transporter 2 (VMAT2), which partakes in vesicular DA storage before carrying its liberation through the nerve cell junction. As a consequence, Cu^II^atsm can not merely escalate the generation of DA, but also situate it within the region needed for effective conveyance.

Further, targeting the profusion in the loading of Fe detected inside the SN-PC region of the brain appears as another propitious approach in the therapy of PD. DFO, DFP, and DFX, pertaining to the family of Fe chelators, have been elucidated to yield promising outcomes in individuals experiencing PD via significantly lowering the clinical manifestations, such as the generation of OH and forfeiture of DArgic nerve cells [183]. The trio, namely, DFO, DFP, and DFX, have procured authorization from the Food and Drug Administration (FDA) for the therapy of a grievous type of β-thalassemia, namely, thalassemia major (TM), and are reported to be highly tolerated [235,236]. DFP has been immensely examined, utilizing cellular and experimental animal models, and pilot clinical studies on human beings, in order to detect its pharmacological aptitude [182,183,237,238]. It has been reported that DFP permeates across the BBB and exerts slight consequences on Fe concentrations within the body and blood parameters, owing to its aptitude to carry out the conveyance of metal chelates to the protein unbound to Fe, namely, apo-Tf [239]. The aptitude of DFP to carry out the scavenging of mobile/fluctuating Fe culminates in the suppression of ROS generation [238,239]. Furthermore, DFP was found to safeguard nerve cells from MPTP in the SH-SY5Y cell line [182] and de-escalate the DArgic nerve cell deprivation and elevate the DA levels inside the SN-PC of 6-OHDAPD model [183]. Pilot clinical studies on human beings employing DFP have demonstrated successful and incentivizing outcomes [237]. The MRI test has detected that DFP exhibits the tendency to minimize the concentrations of Fe in certain areas of the brain, for instance, the dentate nucleus and caudate nucleus, without depleting/eliminating the overall Fe concentrations from the brain. Besides, it has been elucidated that therapy with the aid of DFP contributed to enhancement in motor operations in individuals experiencing PD [237]. These findings might open neoteric and futuristic doors for further research that will look at the DFP optimal therapeutic dose and time period of treatment.

## 8. Conclusions

Parkinson’s disease is a complicated, progressive, age-associated, and multifaceted neurodegenerative malady that arises following the DArgic nerve cell deterioration inside the SN-PC. The etiopathogenesis of PD is still perplexing and abstruse. However, genetic mutations, environmental toxin exposure, and several pathogenic events in amalgamation have been elucidated to be actively engaged in the evolution of the malady. Despite the intricate, perplexing, and multifarious nature of the PD, the pivotal hallmarks, namely, the OS and oligomerization or misfolding of critical protein (α-synuclein), are strongly associated with escalated toxicity to the nerve cells. Redox-active metals, in particular Fe and Cu, are hypothesized to partake in the regulation of pivotal operations of the CNS, such as the synthesis of neurotransmitters, generation of myelin, synaptic signaling, and conveyance of O_2_, but their extortionate buildup in the body culminates in nerve cell toxicity. From the last 10 years, a plethora of investigation has concentrated on deciphering the role of Fe and Cu in nerve cell degeneration, which tends to be multifarious. However, expanding corroboration has suggested that Fe and Cu accumulation in the CNS culminates in the elevation of OS (via the ROS-mediated pathway), α-synucelin aggregation within the LBs, and lipid peroxidation, which as a result contributes to the destruction of DNA and DArgic nerve cell degeneration in the SN-PC. Most notably, a wide range of preclinical and clinical investigations have revealed that metal complexes and metal chelators hold the aptitude to de-escalate nerve cell deterioration and render complete alleviation of the manifestations and cessation of the disease progression, and might emerge as a propitious strategy in the therapy of PD.

## Data Availability

Not applicable.

## Abbreviations

AA, amino acids; AD, Alzheimer’s disease; ALS, amyotrophic lateral sclerosis; apo-Tf, apo-transferrin; APP, amyloid precursor protein; ATOX1, antioxidant 1 Cu chaperone; ATP, adenosine triphosphate; ATP7A, ATPase Cu-transporting alpha; ATP7B, ATPase Cu-transporting beta; BBB, blood–brain barrier; BCECs, brain capillary endothelial cells; C, carboxy; Ca, calcium; Ca^2+^, Ca ion; CA, catecholamine; CaN, calcineurin; CAT, catalase; CCl_4_, carbon tetrachloride; CCS, Cu chaperone for SOD1; CNS, central nervous system; Co, cobalt; COMT, catechol-O-methyltransferase; Cox17, Cu chaperone for cytochrome c oxidase; CP, ceruloplasmin; CSF, cerebrospinal fluid; CTR1, Cu-transporter 1; Cu, copper; Cu^II^atsm, Cu complexed diacetyl-bis (4-methylthiosemicarbazonato) Cu^II^; Cu^+1^, cuprous form of Cu; Cu^+2^, cupric form of Cu; CuSO_4_, Cu sulphate; CyPA, cyclophilin A; DA, dopamine; DAQ, DA quinone; DArgic, dopaminergic; DASQ, DA semiquinone radical; DFO, desferrioxamine; DFP, deferiprone; DFX, deferasirox; *DJ-1*, *protein deglycase*; DMT1, divalent metal transporter 1; DNA, deoxyribonucleic acid; DOHH, deoxyhypusine hydroxylase; DOPAC, 3,4-dihydroxyphenylacetic acid; ETC, electron transport chain; FDA, Food and Drug Administration; Fe, iron; Fe^+2^, ferrous Fe; Fe^+3^, ferric Fe; Fe^+4^, ferryl Fe; FPN1, ferroportin 1; GABA_A_, γ-aminobutyric acid A; *GBA*, *glucocerebrosidase*; GSH, glutathione; Hb, haemoglobin; Hg, mercury; HIF, hypoxia-inducible factor; HO-1, heme oxygenase-1; holo-Tf, holo-transferrin; H_2_O_2_, hydrogen peroxide; IL-1, interleukin-1; IREs, Fe-responsive elements; IRPs, Fe regulatory proteins; K, potassium; LBs, Lewy bodies; LC, locus coeruleus; L-dopa, Levodopa; Lf, lactoferrin; *LRRK2*, *leucine-rich repeat kinase 2*; MAO-B, monoamine oxidase B; MDA, malondialdehyde; Mg, magnesium; Mn, manganese; MNK, Menkes protein; MPP^+^, 1-methyl-4-phenylpyridinium ion; MPTP, 1-methyl-4-phenyl-1,2,3,6-tetrahydropyridine; MRI, magnetic resonance imaging; mRNA, messenger ribonucleic acid; MTs, metallothioneins; Na, sodium; NADH, nicotinamide adenine dinucleotide hydrogen; NF, neurofilaments; Ni, nickel; NM, neuromelanin; NMDA, N-methyl D-aspartate; NO, nitric oxide; NO_2_, nitro; NOS, NO synthase; NSAIDs, non-steroidal anti-inflammatory drugs; NTBI, non-Tf bound Fe; O_2_, oxygen; O_2_^−^, superoxide; OH, hydroxyl radicals; 6-OHDA, 6-hydroxy DA; ONOO^−^, peroxynitrite; OS, oxidative stress; *Parkin*, *Parkin RBR E3 ubiquitin-protein ligase*; Pb, lead; PCBP, poly r(C)-binding protein; PD, Parkinson’s disease; PERC, perchloroethylene; PET, positron emission tomography; pH, potential hydrogen; PH, prolyl hydroxylase; *PINK1*, *PTEN-induced kinase 1*; PP2B, protein phosphatase 2B; PUFAs, polyunsaturated fatty acids; RBCs, red blood cells; ROS, reactive oxygen species; S, sulphur; SH-SY5Y, human neuroblastoma cells; SN, substantia nigra; SN-PC, substantia nigra pars compacta; SOD, superoxide dismutase; STEAP3, six-transmembrane epithelial antigen of prostate 3; TCE, trichloroethylene; Tf, transferrin; TfR1, Tf receptor 1; TH, tyrosine hydroxylase; TM, thalassemia major; *UCHL1*, *ubiquitin carboxy (C)-terminal hydrolase L1*; UPS, ubiquitin-proteasome system; 5′-UTR, 5′-untranslated region; VMAT2, vesicular monoamine transporter 2; *VPS35*, *vacuolar protein sorting 35*; WND, Wilson disease protein; ZIP, ZRT/IRT-like proteins; Zn, Zinc.
